# Supplementary figures and images for: Box C/D small nucleolar ribonucleoproteins regulate mitochondrial surveillance and innate immunity
Source: PLoS Genet. 2022 Mar 11;18(3):e1010103. doi: 10.1371/journal.pgen.1010103 (PMC8942280; doi:10.1371/journal.pgen.1010103)

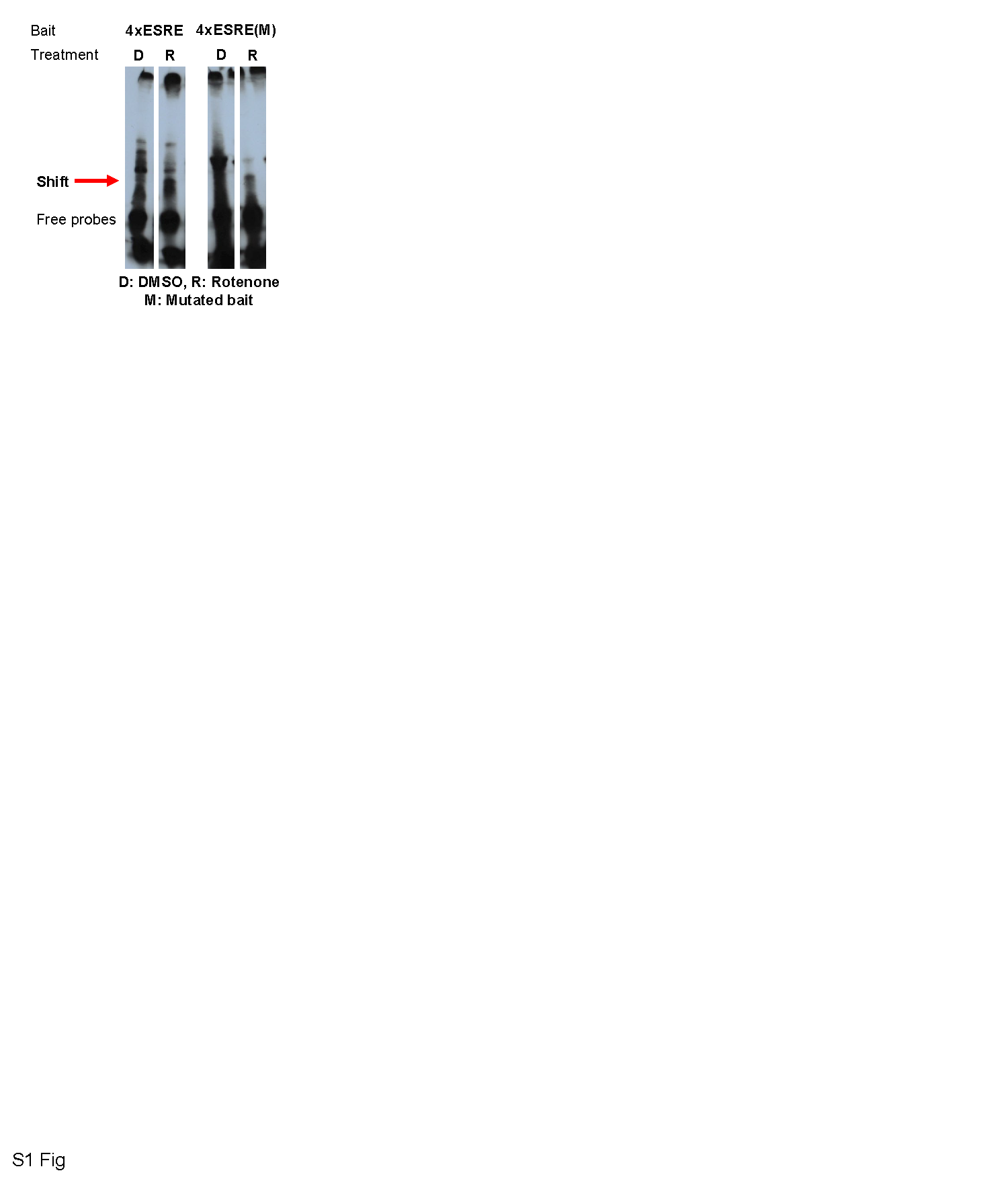

Supplement: S1 Fig — Electrophoretic mobility shift assay (EMSA) showed the presence of the ESRE-binding motif through the identified ‘Shift’. Worms were treated with DMSO or 50 μM rotenone for 14 hours. D: DMSO-treated, R: rotenone-treated, 4xESRE: oligo bait, and 4xESRE(M): oligo bait with mutations. (TIFF) [file pgen.1010103.s001.tiff]

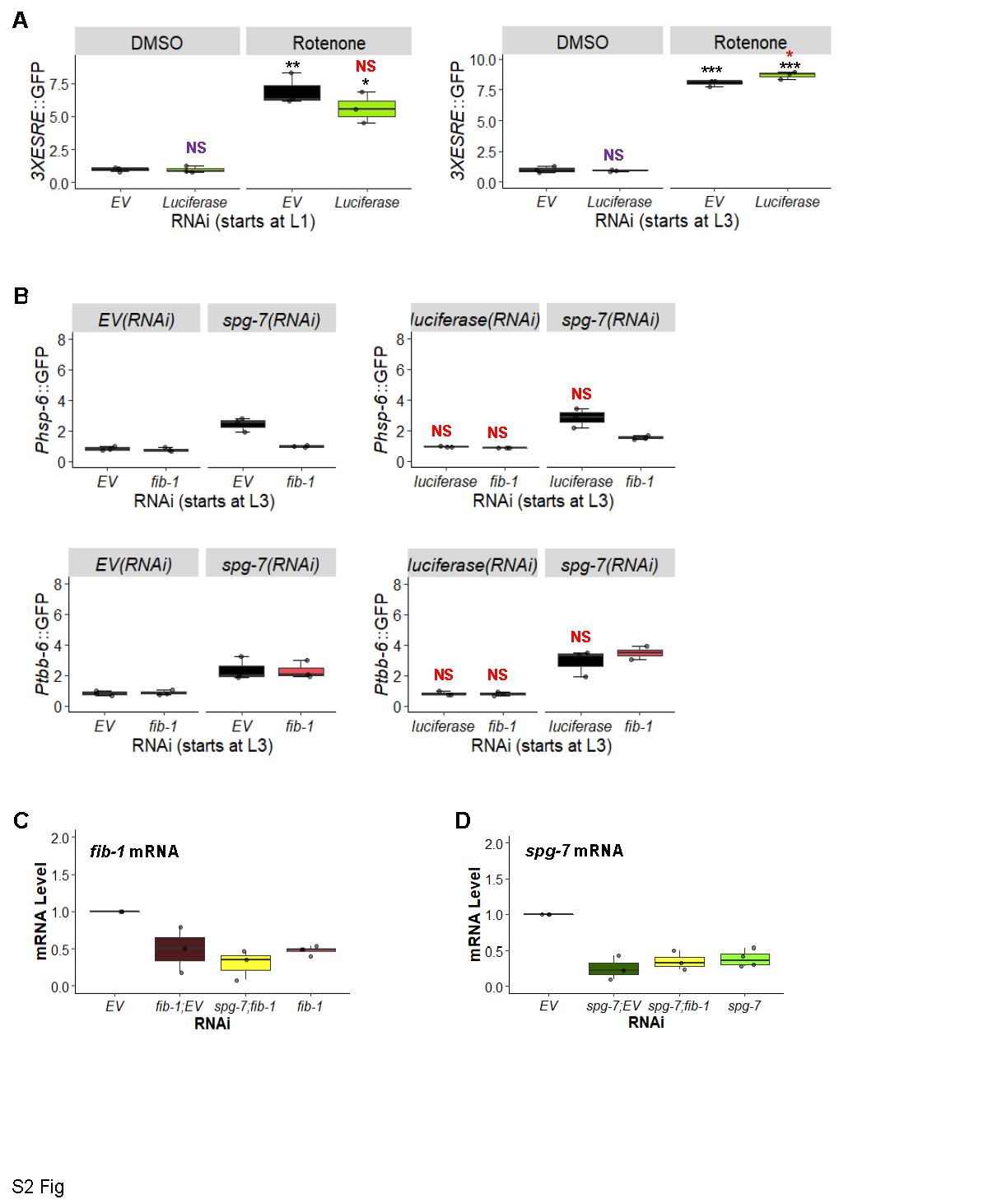

Supplement: S2 Fig — (A) Quantification of GFP fluorescence of C. elegans carrying 3XESRE::GFP that were reared on E. coli expressing RNAi targeting empty vector (EV) or luciferase. Worms were treated for 8 hours with vehicle (DMSO) or 25 μM rotenone. RNAi was started at L1 (left) or L3 stage (right). (B) Quantification of GFP fluorescence of C. elegans carrying Phsp-6::GFP (top) and Ptbb-6::GFP (bottom) reporters that were reared on E. coli expressing RNAi targeting empty vector (EV) or luciferase. Double RNAi was performed with empty vector (EV) or luciferase, or spg-7(RNAi). Three biological replicates with ~400 worms/replicate were analyzed. p-values were determined from Student’s t-test. GFP values were normalized to EV-DMSO or EV. NS not significant, *p < 0.05, ** p < 0.01, *** p < 0.001. In (A), purple significance marks indicate comparison of luciferase(RNAi) to EV(RNAi) in DMSO, red marks indicate comparison of luciferase(RNAi) to EV(RNAi) in rotenone, and black marks indicate comparison between expressions in rotenone and DMSO. In (B), red significance marks indicate comparison between luciferase(RNAi) to the corresponding condition but with EV(RNAi), e.g., fib-1;luciferase(RNAi) vs. fib-1;EV(RNAi) and spg-7;luciferase(RNAi) vs spg-7;EV(RNAi). (TIF) [file pgen.1010103.s002.tif]

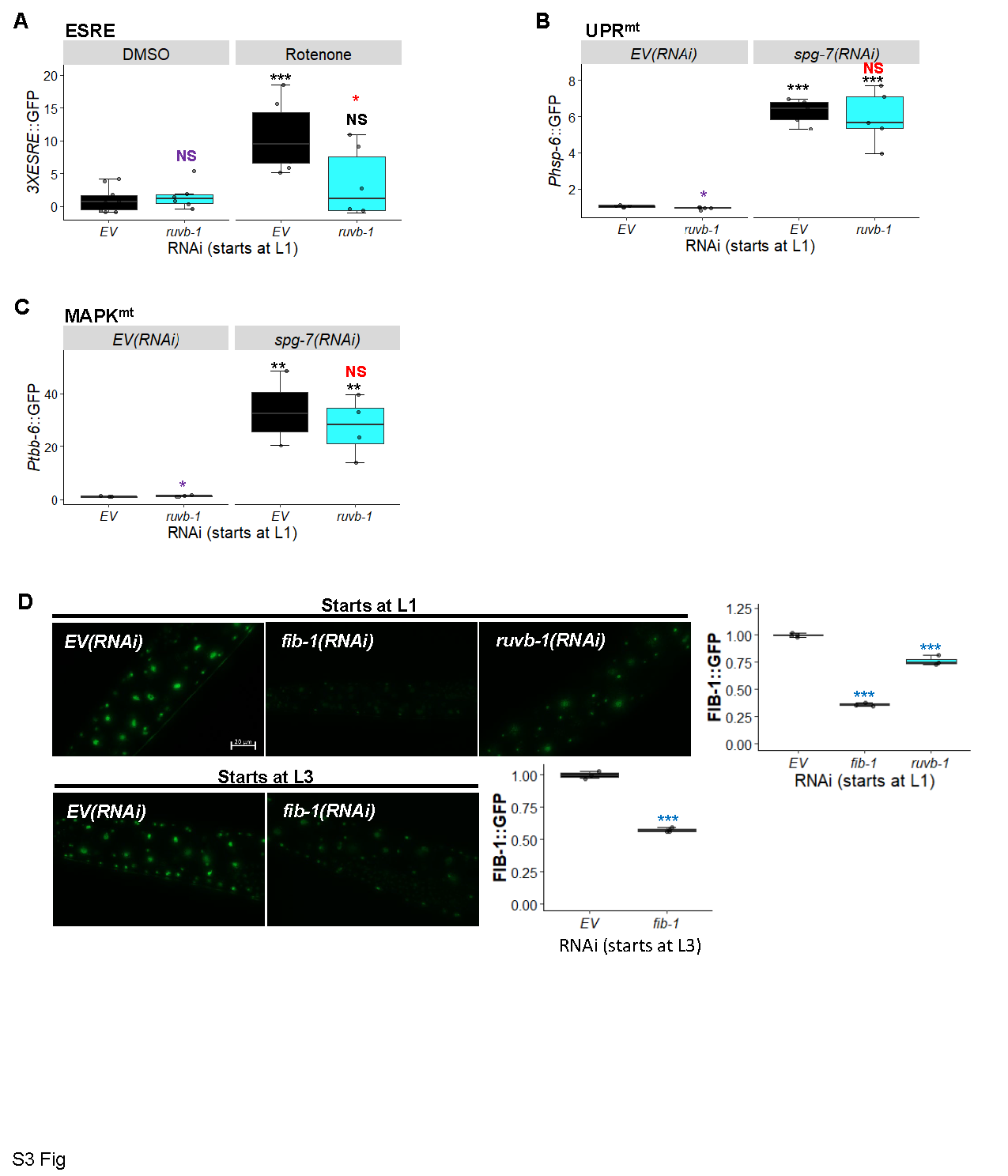

Supplement: S3 Fig — Quantification of GFP fluorescence of C. elegans carrying (A) 3XESRE::GFP, (B) Phsp-6::GFP, and (C) Ptbb-6::GFP reporters that were reared on E. coli expressing RNAi targeting empty vector (EV) or ruvb-1/RUVB. In (A), worms were treated for 8 hours with vehicle (DMSO) or 50 μM rotenone. In (B, C), double RNAi was performed with empty vector (EV) or spg-7(RNAi). (D) Images and fluorescence intensity quantification of C. elegans carrying Pfib-1::FIB-1::eGFP reporter that were reared on E. coli expressing RNAi targeting empty vector (EV), fib-1/FBL, or ruvb-1/RUVB. RNAi was started at L1 or L3 stage as indicated in the figure. Three biological replicates with (A-C) ~400 worms/replicate or (D) ~25 worms/replicate were analyzed. p-values were determined from Student’s t-test. GFP values were normalized to EV-DMSO or EV. NS not significant, *p < 0.05, ** p < 0.01, *** p < 0.001. In (A-C), purple significance marks indicate comparison of ruvb-1(RNAi) to EV(RNAi) in unstressed condition (DMSO or EV(RNAi)), red marks indicate comparison of ruvb-1(RNAi) to EV(RNAi) in stressed condition (rotenone or spg-7(RNAi)), and black marks indicate comparison between stressed and unstressed conditions. (TIFF) [file pgen.1010103.s003.tiff]

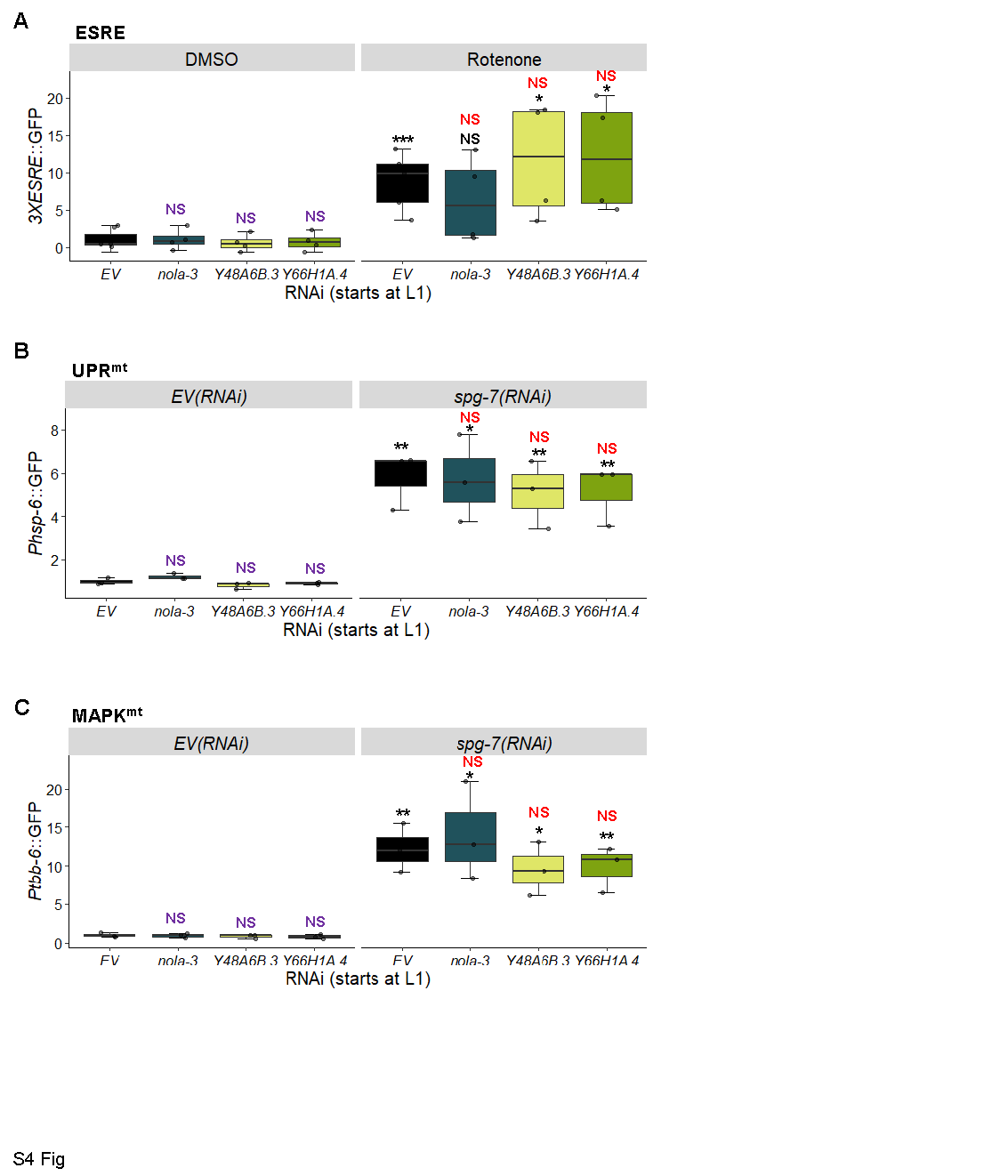

Supplement: S4 Fig — Quantification of GFP fluorescence of C. elegans carrying (A) 3XESRE::GFP, (B) Phsp-6::GFP, or (C) Ptbb-6::GFP reporters that were reared on E. coli expressing empty vector (EV) or RNAi targeting box H/ACA snoRNP members: nola-3/Nop10, Y48A6B.3/Nhp2, or Y66H1A.4/Gar1. In (A), worms were treated for 8 hours with vehicle (DMSO) or 50 μM rotenone. In (B, C), double RNAi was performed with empty vector (EV) or spg-7(RNAi). Three biological replicates with ~400 worms/replicate were analyzed. p-values were determined from two-way ANOVA, followed by Dunnett’s test, and Student’s t-test. All fold changes were normalized to DMSO-EV or EV control. NS not significant, *p < 0.05, ** p < 0.01, *** p < 0.001. In all panels, purple significance marks indicate comparison of H/ACA genes(RNAi) to EV(RNAi) in unstressed condition (DMSO or EV(RNAi)), red marks indicate comparison of H/ACA genes(RNAi) to EV(RNAi) in stressed condition (rotenone or spg-7(RNAi)), and black marks indicate comparison between stressed and unstressed conditions. (TIF) [file pgen.1010103.s004.tif]

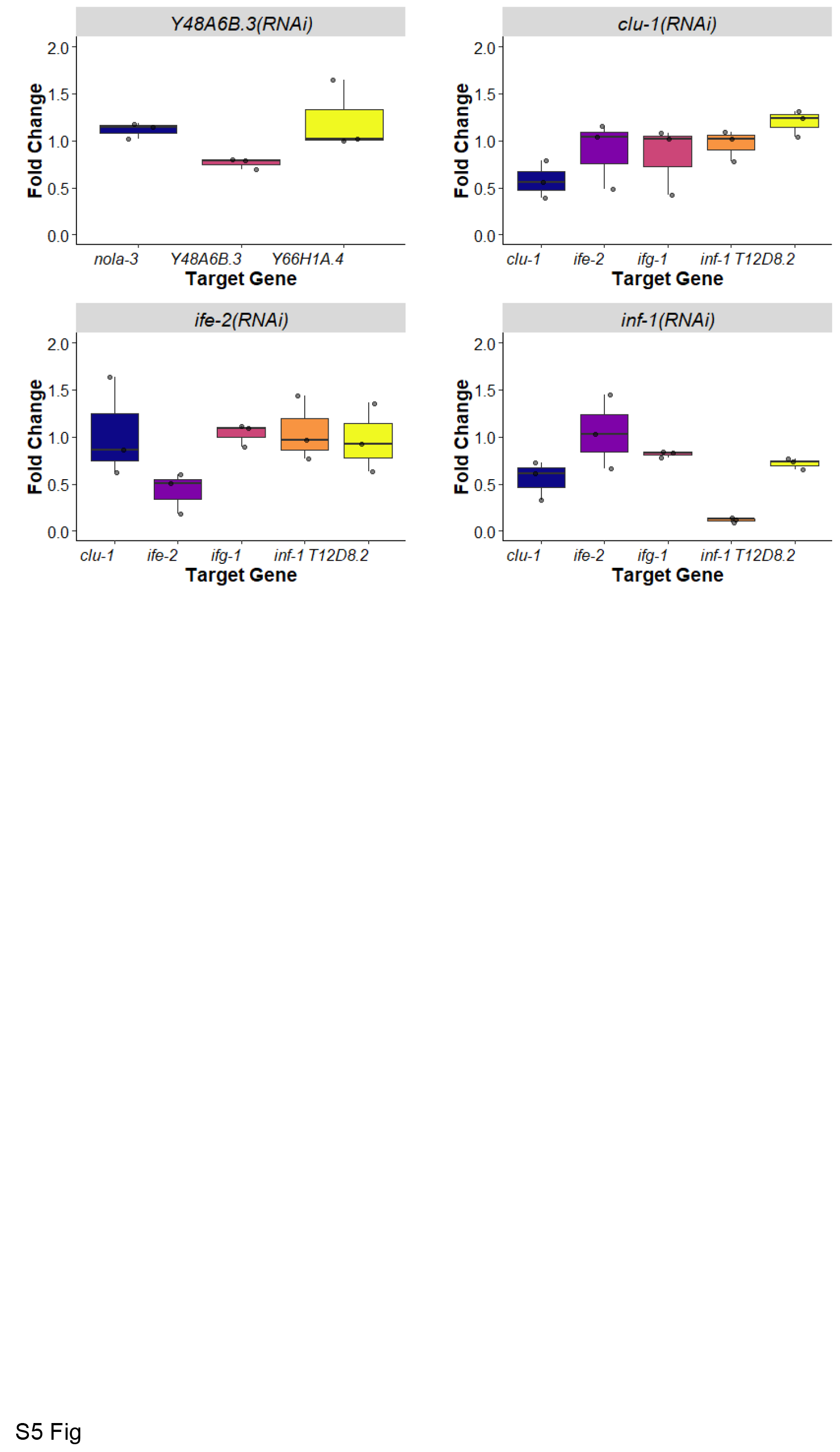

Supplement: S5 Fig — mRNA levels of target genes in N2 worms reared on E. coli expressing RNAi targeting Y48A6B.3, clu-1/eIF3A, ife-2/ eIF4E, or inf-1/eIF4A. Three biological replicates with ~8,000 worms/replicate were analyzed. All fold changes were normalized to expression of target genes in worms reared on EV(RNAi)-expressing E. coli. (TIF) [file pgen.1010103.s005.tif]

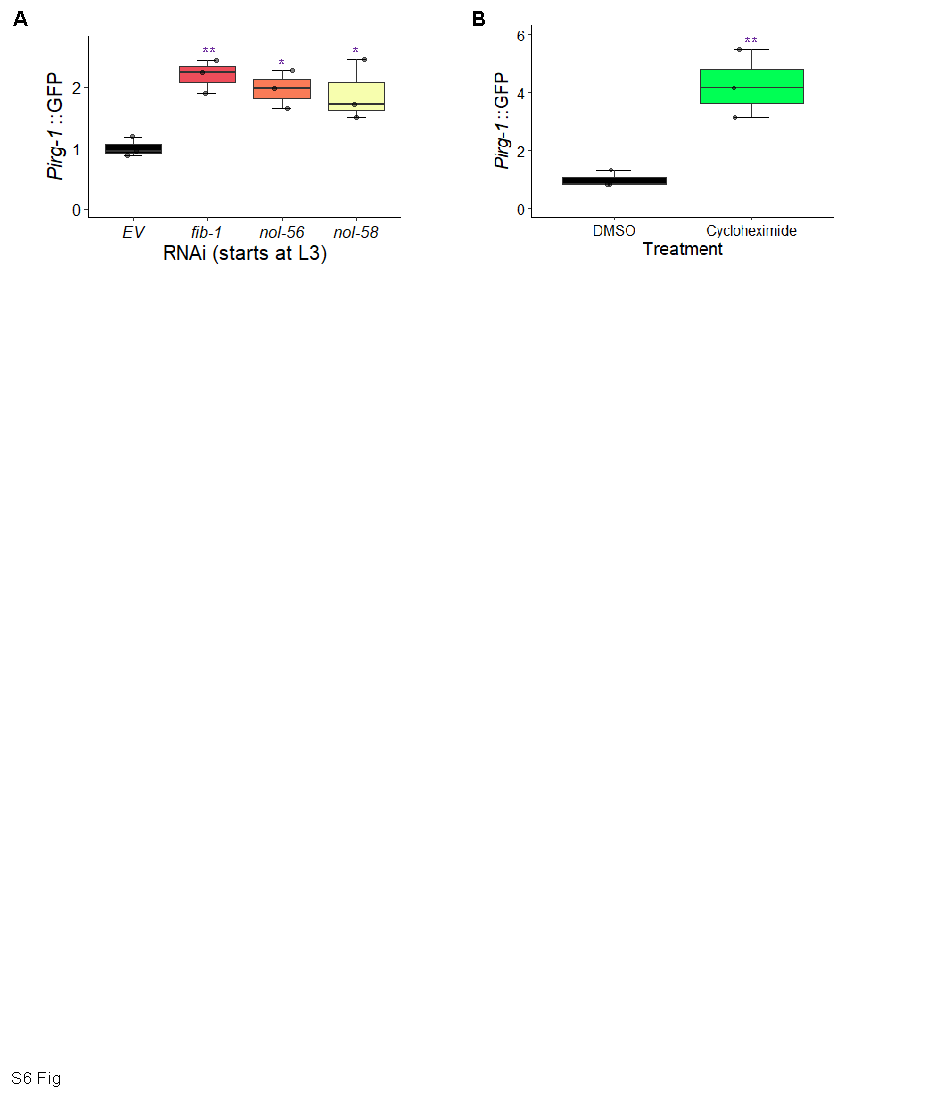

Supplement: S6 Fig — (A, B) Quantification of GFP fluorescence of C. elegans carrying Pirg-1::GFP reporter that (A) were reared on E. coli expressing empty vector (EV), fib-1(RNAi), nol-56(RNAi), or nol-58(RNAi) or (B) exposed to 2 mg/mL cycloheximide or DMSO control for 8 hours. Three biological replicates with ~400 worms/replicate were analyzed. p-values were determined from (A) one-way ANOVA, followed by Dunnett’s test or (B) Student’s t-test. All fold changes were normalized to EV or DMSO control. *p < 0.05, ** p < 0.01. (TIF) [file pgen.1010103.s006.tif]

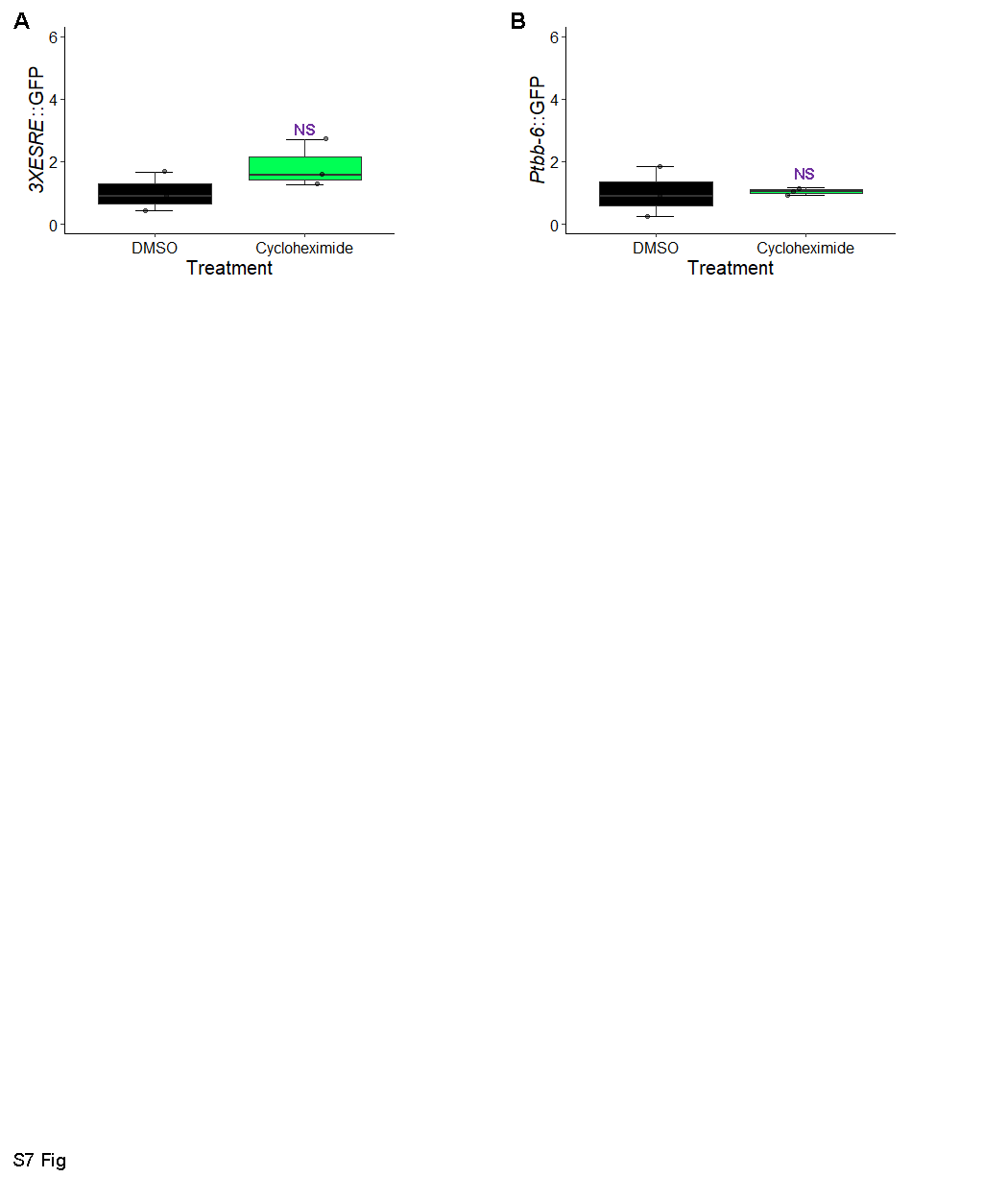

Supplement: S7 Fig — Quantification of GFP fluorescence of C. elegans carrying (A) 3XESRE::GFP or (B) Ptbb-6::GFP reporters that were treated for 8 hours with vehicle (DMSO) or 2 mg/mL of translation elongation inhibitor cycloheximide. Three biological replicates with ~400 worms/replicate were analyzed. p-values were determined from Student’s t-test. NS not significant. (TIF) [file pgen.1010103.s007.tif]

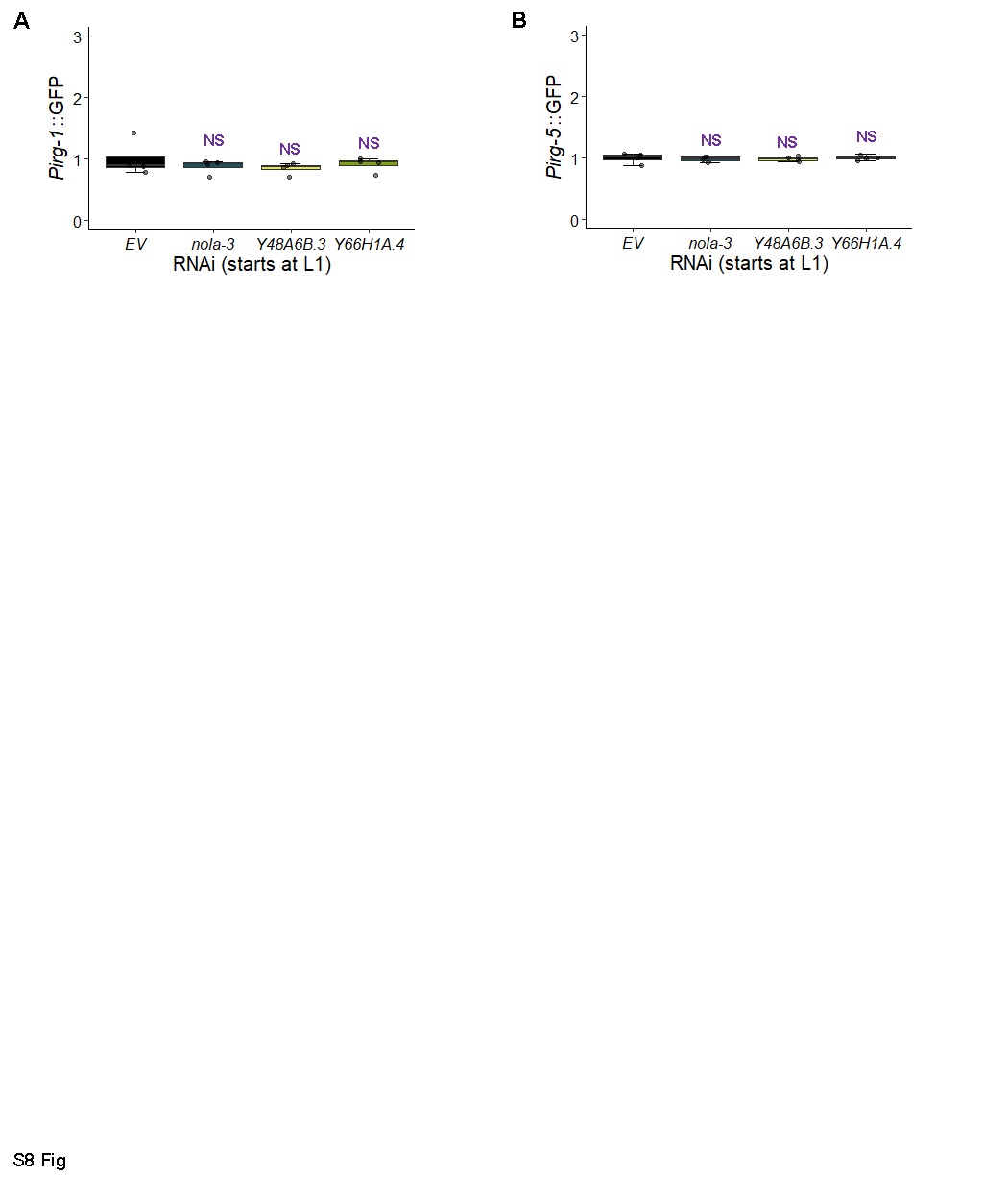

Supplement: S8 Fig — Quantification of GFP fluorescence of C. elegans carrying (A) Pirg-1::GFP or (B) Pirg-5::GFP reporters that were reared on E. coli expressing empty vector (EV) or RNAi targeting box H/ACA snoRNP members: nola-3/Nop10, Y48A6B.3/Nhp2, or Y66H1A.4/Gar1. Three biological replicates with ~400 worms/replicate were analyzed. p-values were determined from one-way ANOVA, followed by Dunnett’s test. All fold changes were normalized to EV control. NS not significant. (TIF) [file pgen.1010103.s008.tif]

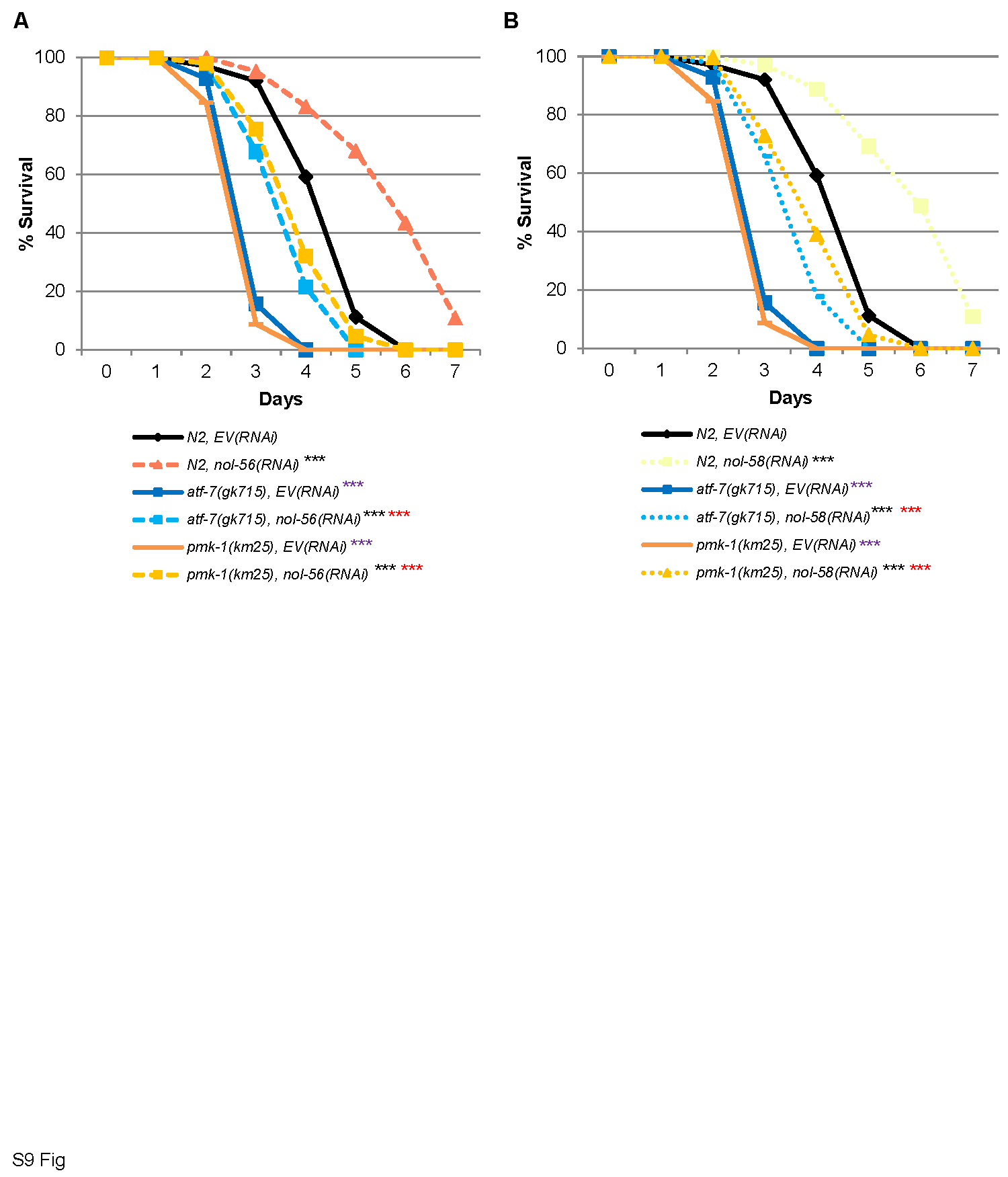

Supplement: S9 Fig — (A, B) Survival of N2 wild-type, atf-7(gk715), or pmk-1(km25) mutants reared on RNAi strains targeting empty vector (EV) and (A) nol-56 or (B) nol-58. Three biological replicates with ~150 worms/replicate were analyzed. Representative replicates are shown. p-values were determined from log-rank test. *** p < 0.001. Purple significance marks indicate comparison of atf-7(gk715) or pmk-1(km25) mutants to N2 wild-type (reared on EV(RNAi)), red marks indicate comparison of atf-7(gk715) or pmk-1(km25) mutants to N2 wild-type (reared on nol-56(RNAi) or nol-58(RNAi)), and black marks indicate comparison between nol-56(RNAi) or nol-58(RNAi) vs. EV(RNAi) for each worm strain. (TIF) [file pgen.1010103.s009.tif]
