# Supplementary material for: Box C/D small nucleolar ribonucleoproteins regulate mitochondrial surveillance and innate immunity
Source: PLoS Genet. 2022 Mar 11;18(3):e1010103. doi: 10.1371/journal.pgen.1010103 (PMC8942280; doi:10.1371/journal.pgen.1010103)
Supplement: Attachment — Submitted filename: Tjahjono et al Author Response.pdf [file pgen.1010103.s016.pdf]

Dear Editor and Reviewers,

Please find our point-by-point responses to the reviewers' comments below. Amendments to the manuscript that involved experiments or additional analysis are indicated in red, while clarifications of terms, typos, etc. are in blue.

Reviewer #1

The manuscript by Tjahjono, Revtovich, and Kirienko describe a new role for the box C/D small nucleolar ribonucleoproteins in regulating mitochondrial recovery and innate immunity. The authors wished to identify new regulators of the ESRE network, a mitochondrial surveillance pathway that comprises genes whose promoters contain the ESRE motif. Using a biochemical pulldown approach involving a tandem ESRE motif repeat oligonucleotide, the authors identified the box C/D snoRNP complex members FIB-1 and NOL-56, as regulators of the ESRE pathway. In addition, box C/D snoRNPs also regulated other mitochondrial surveillance pathways including the UPR^{mt} and $MAPK^{mt}$. The spatial requirement of the snoRNP in regulating these mitochondrial surveillance pathways was also examined using RNAi against ruvb-1, a component required for assembly and localization of box C/D snoRNP to the nucleoli. Nucleolar localization of snoRNP was required for ESRE regulation but only partially for the $MAPK^{mt}$ pathway and was not required for the UPR^{mt}. The authors also confirmed that members of the H/ACA snoRNP complex do not have a role in mitochondrial surveillance and furthermore, that defects in translation are unlikely the cause for the effects observed with box C/D snoRNP knockdown. Lastly, using previously established reporters, the authors find that box C/D snoRNP functions to repress the innate immune response. Consistent with this finding, knockdown of members of the box C/D snoRNP increases host survival during infection with *P. aeruginosa* under slow-killing conditions in which the host succumbs to pathogen colonization. However, loss of box C/D snoRNP members rendered animals more sensitive to *P. aeruginosa* under liquid-killing conditions, which kills the host via iron sequestration from mitochondria. The manuscript is overall a well-written body of work and the identification of the box C/D snoRNP complex as a regulator of mitochondrial surveillance and host immunity is novel and will be of interest to those studying cellular stress response pathways and host-pathogen interactions. However, the following suggestions could help improve the author's findings.

Major revisions

The authors' model suggests that box C/D snoRNPs encourage mitochondrial surveillance pathways by suppressing the immune response which can be quite energy-consuming. However, these conclusions are based on a handful of specific gene transcriptional GFP reporters and limited survival/lifespan assays. I would suggest the following to make their model more substantive.

1. The authors suggest that loss of box C/D snoRNPs reduces mitochondrial recovery, a finding that is based on altered activity of mitochondrial surveillance reporters. The authors should assay mitochondrial functions in the absence of box C/D snoRNPs such as mitochondrial membrane potential and/or oxygen consumption rates.

We appreciate the reviewer's point, and agree that these measurements would provide a more direct argument of the importance of the box C/D snoRNPs in mitochondrial function. We have performed

measurements of mitochondrial membrane potential, mitochondrial mass, and oxygen consumption rate in wild-type worms reared on E. coli targeting the box C/D snoRNPs, as compared to the empty vector control. Mitochondrial membrane potential was measured by using MitoTracker Red, a dye that accumulates in the mitochondria dependent on their potential, while mitochondrial mass was measured by using MitoTracker Green, a dye that is comparatively insensitive to membrane potential. Finally, oxygen consumption rate was measured using a classical technique, via a biological oxygen monitor with a Clark-type oxygen electrode. Consistent with our model, we observed reduction in all three parameters. The resulting graphs are incorporated into the new figure panels ***Figure 9A-F****. However, it should be noted that we attribute the decrease in mitochondrial membrane potential and oxygen consumption rate to a proportional decrease in mitochondrial mass. Again, our model predicts that disruption of the C/D snoRNPs dysregulates mitochondrial surveillance pathways, and compromises normal mitochondrial biology.*

2. It is intriguing that loss of *fib-1*, but not other box C/D snoRNPs complex members, results in increased lifespan (Figure 1E) since mitochondrial surveillance is predicted to be compromised in this animal. Mitochondrial stress is a known inducer of increased longevity, as observed with mitochondrial mutants *clk-1*, *isp-1*, *nuo-6* or *cco-1* RNAi, among others. The UPR^{mt} and $MAPK^{mt}$ are also required for the extended lifespan of some of these conditions. It would be interesting to measure the lifespan for some of these long-lived mitochondrial stressed animals in the presence or absence of FIB-1. If FIB-1 is truly promoting mitochondrial surveillance, then the increased lifespan of *fib-1* RNAi animals should not be additive with the increased lifespan of these mitochondrial stressed animals. Instead, *fib-1* RNAi should reduce the lifespans of these animals (possibly to levels observed with *fib-1* RNAi alone) based on the authors' model, due to their compromised mitochondrial surveillance ability.

We thank the reviewer for this suggestion. We would note, however, that lifespan experiments (while very interesting for what they represent) are a single phenotype that is the readout of potentially dozens to hundreds of pathways with varying impact. As such, they may not represent an ideal readout.

Nevertheless, we performed the suggested experiments using two well-known long-lived mitochondrial Mit mutants: nuo-6(qm200) and isp-1(qm150). Each strain was reared on E. coli expressing either empty vector (EV) RNAi or fib-1(RNAi). Unfortunately, after genotyping our nuo-6(qm200) mutant stock, we discovered that it was the wrong strain.

Worms were grown on NGM plates containing 5-fluoro-2'-deoxyuridine (FuDR) to eliminate the potential confound of offspring in this self-fertile model. The use of this compound also eliminates the need to repeatedly transfer worms from plate to plate to avoid offspring, which can also introduce a stochastic factor (due to physical damage during transfer) that can complicate lifespan analysis. In the process of this research, we discovered an unexpected sensitivity of N2 worms reared on fib-1(RNAi) to FuDR exposure; starting at day 12, N2 worms reared on fib-1(RNAi) began to die much more rapidly than the empty vector cohort. The explanation for this sensitivity was neither immediately clear nor was a potential explanation available in the literature. FuDR is often used in C. elegans research to ensure worm sterility. This has led to a variety of experimental observations, which have included both ***increased*** *lifespans (often argued to arise from the release of the metabolic demand of reproduction) (1, 2) but also* ***decreased*** *lifespans (perhaps due to DNA damage from this compound, which is a nucleotide analog) (3). As a consequence of these outcomes, it was not possible to directly address the question raised by the reviewer in the timeframe available.*

It is worth noting that the addition of FuDR did not apparently negatively impact survival of worms reared on fib-1(RNAi) on the agar pathogenesis assay (Slow Killing), as will be discussed further below. One possible explanation is that this assay is shorter in duration (~7 days compared to ~21-28 days for longevity). Alternatively, it is possible that this effect was smaller in scale than the increased survival provided by fib-1(RNAi) in that assay. In that case, a truer measure would reveal an even greater effect of fib-1(RNAi) on survival if not for the consequences of FuDR exposure.

3. Related to comment 2) the authors did not observe increased lifespan in the absence of nol-56/58. Is this a consequence of stronger RNAi knockdown of these genes compared to fib-1 RNAi? If *nol-56/58* RNAi were to be diluted, would they observe an increase in lifespan or is this effect specific to *fib-1* RNAi?

We thank the reviewer for the suggestion, it is certainly an interesting idea. We attempted to determine whether this was the case by diluting the RNAi two- or four-fold and testing the influence of these conditions on lifespan. In this case, we did not observe any significant difference amongst the RNAi dilution conditions on the magnitude of the extension of lifespan conferred by fib-1(RNAi). We also did not see any reliable effect of the dilutions of both nol-56(RNAi) and nol-58(RNAi). Because we were not able to see any consistent or meaningful difference, we consider this outcome is probably not worth reporting in the paper, but is attached below.

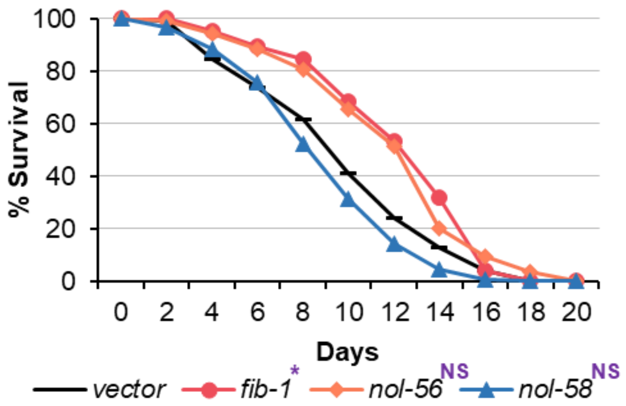

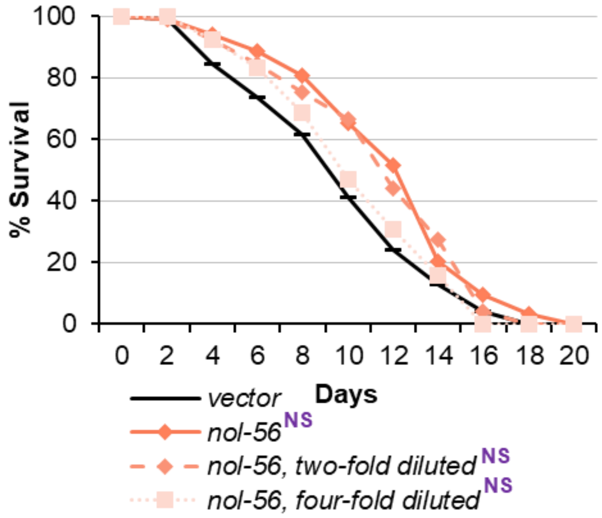

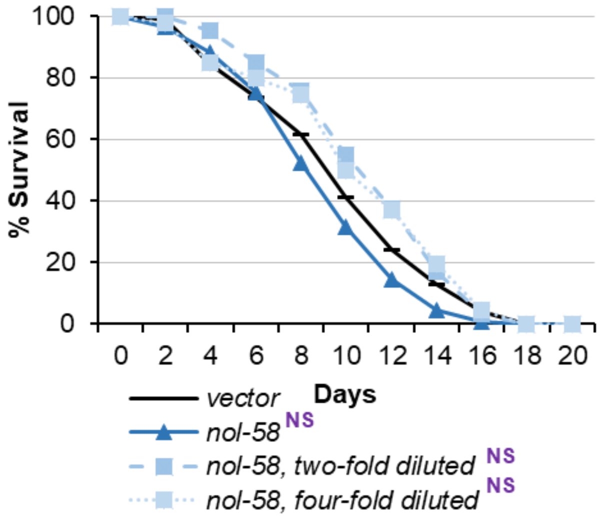

4. a) The author's model proposes that the box C/D snoRNPs repress immunity to favor mitochondrial surveillance. They show that two immune reporters, *irg-1*::GFP and *irg-5*::GFP are induced with box C/D snoRNP RNAi. Ideally, a more global view of transcriptional changes occurring in the presence or absence of box C/D snoRNP using RNAseq would provide more support for their model. Alternatively, one could test by qPCR a set of immune genes that are known to be regulated by mitochondrial surveillance programs (e.g. Campos et al. 2021 PMID 34617666, Pellegrino et al. 2014; PMID 25274306) in the presence or absence of box C/D snoRNPs.

We appreciate the point that the reviewer is making. Although it is somewhat common to use a small set of reporters to test the outcome of immune disruption, it is a fair point that immune responses can vary significantly, and that the immune response may vary significantly under different conditions. We agree that transcriptional quantification of a broader set of genes would strengthen our conclusions.

*To address this, we performed qRT-PCR of eight immune genes that are regulated by mitochondrial surveillance (4), as suggested. We found that all these immune genes were induced by the loss of FIB-1. We also verified the induction of irg-1, irg-5, and irg-2, via qRT-PCR after treatment with fib-1(RNAi). These data have been added as the new panel **Figure 6E**, where dark blue bars represent immune genes regulated by mitochondrial surveillance programs, and light blue bars indicate other immune genes.*

b) Also, pathogen gut colonization should be tested to complement their slow-killing survival assay and to further support their model that immune responses and host resistance are enhanced with loss of box C/D snoRNP.

*The reviewer makes a very good point here. The connection between survival and colonization is complex. We have quantified intestinal colonization under the same assay conditions as described. As expected, the loss of Box C/D snoRNPs resulted in lower colonization of the intestine (**Figure 8C**). We think that the likeliest explanation for this is the increased host immune response previously described.*

5. The authors find that transcriptional induction of immune genes following loss of box C/D snoRNP is partially or fully dependent on PMK-1/p38 and ATF-7/ATF7. The authors should perform their slow-killing survival assays in the presence or absence of PMK-1/ATF-7 to support their gene expression data. Also, is ZIP-2 implicated in the increased survival of box C/D snoRNPs deficient animals? This seems relevant since irg-1 was induced in the absence of box C/D snoRNP and ZIP-2 is a critical regulator of this gene during *P. aeruginosa* infection and with translation repression. As well, the authors previously determined ZIP-2 to be related to ESRE gene expression regulation.

The reviewer is, of course, quite correct regarding the importance of the PMK-1 MAPK pathway in the response to intestinal colonization, as is well-established in the C. elegans field. Likewise, ZIP-2 plays an important role in the response to infection with P. aeruginosa.

*As such, we performed Slow Killing assays with atf-7(gk715) and pmk-1(km25) mutants and compared the outcome to infection of N2 worms. Interestingly, the loss of Box C/D snoRNPs increased survival of atf-7(gk715) and pmk-1(km25) mutants, as was observed for wild-type worms (**Figure 8D** for fib-1(RNAi) and **Figure S9** for nol-56(RNAi) and nol-58(RNAi)). Similarly, we assayed the involvement of ZIP-2 by using a glp-4;zip-2 mutant and compared it to glp-4. As before, the double mutants appeared to show an increase in survival in the presence of fib-1(RNAi). Unfortunately, the time constraints limited our ability to complete a sufficient number of biological replicates of this experiment to draw reliable conclusions; only one replicate could be completed. Due to this, we cannot formally draw any conclusion regarding the glp-4; zip-2 double mutants, and these data will not be presented in the final version of this manuscript.*

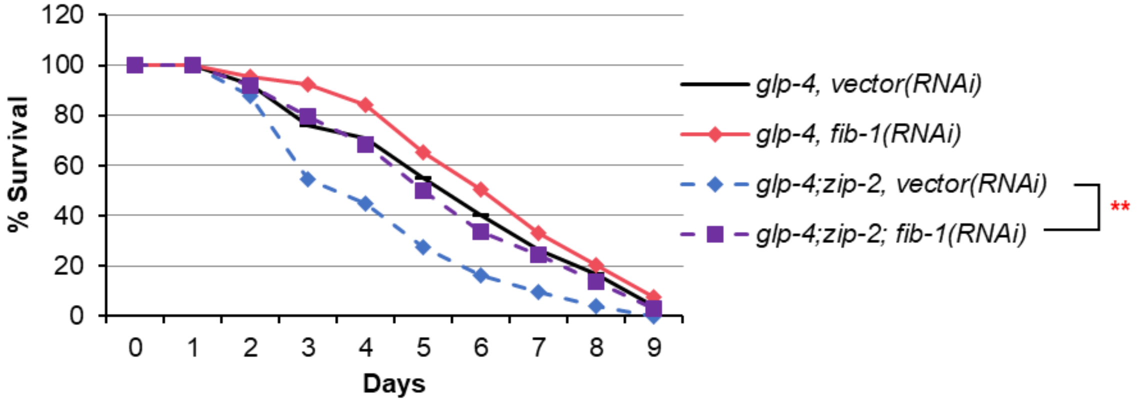

These results are not entirely unexpected. Although the p38 MAPK pathway is very important for host defense against P. aeruginosa, there are multiple immune genes that are not under its control, such as irg-1 and irg-2 (5). Similarly, ZIP-2 is not required for the expression of multiple immune genes; it is not

surprising that the loss of Box C/D snoRNPs still increases survival in the Slow Killing assay. This result strengthens our hypothesis that the Box C/D snoRNPs increased hosts' immune responses globally.

Minor revisions

1. lines 201-208: The authors should speculate why the localization of box C/D snoRNPs is relevant for the regulation of some, but not all, of the mitochondrial surveillance pathways.

Previous research from the Schaffer Lab has found that box C/D snoRNAs from the Rpl13a locus mediate oxidative stress response (6). The snoRNAs travel to the cytoplasm upon exposure to nuclear superoxide. The interference of assembly and localization of snoRNPs had been found to lead to snoRNA loss. As the ESRE pathway is highly responsive to increased superoxide level (7), it is possible that the loss of intermediate snoRNAs (due to mis localization of snoRNPs) will lead to the reduction of ESRE pathway expression. This regulation might be different for the other mitochondrial surveillance pathways less responsive to ROS accumulation. While this explanation is consistent with the observations that we and others have made, it is not directly supported by the data, so we have chosen not to include it in the paper.

2. lines 214-16: The authors should comment further on their finding of increased FIB-1::eGFP punctae size with rotenone. What is the significance of this effect? Also, is the increased size of FIB-1::eGFP punctae specific for rotenone treatment? Do other mitochondrial stress conditions that activate the UPR^{mt}/$MAPK^{mt}$ have a similar effect? Do they also observe increase puncta size during infection with *P. aeruginosa* slow-killing/liquid killing conditions?

We thank the reviewer for this inquiry. In truth, it is quite difficult to determine what the precise significance of this is, which is why we merely made the observation and have not stated that this difference has a mechanistic relevance.

*However, we have performed additional testing with a variety of pharmaceutical agents to see what impact they may have on the size of FIB-1::eGFP punctae. Treatment of worms with compounds known to disrupt mitochondrial function (such as CCCP or rotenone) were seen to increase in the size of FIB-1::eGFP punctae (**Figure 4A**). Additionally, exposure of worms to the mitochondria-damaging toxin pyoverdine (a siderophore produced by the pathogen P. aeruginosa) also increased the size of FIB-1::eGFP punctae (**Figure 8E**). Like the other conditions described here, exposure to pyoverdine is known to result in mitochondrial damage.*

*In contrast, other stress-inducing chemicals (such as the translational inhibitor cycloheximide or the drug tunicamycin) reduced the size of FIB-1::eGFP punctae (**Figure 4B**). Also consistent with other observations, treatment with P. aeruginosa in an agar pathogenesis model (Slow Killing) reduced FIB-1::eGFP punctae size (as compared to E. coli in the same condition) (**Figure 8F**), consistent with previous finding that pathogen exposure reduced both FIB-1 level and nucleolar size (8).*

The most obvious explanation in these cases is that mitochondrial damage leads to increased expression of fib-1 and, in turn, larger punctae. This is consistent with the other observations made in our manuscript.

3. More details on statistical analysis for lifespan and survival assays are needed. A supplementary table listing all survival/lifespan replicates should be provided. Included in this table should be the number of animals censored for their experiments. Also, statistical comparisons for these assays should follow the guidelines found in Petrascheck and Miller (2017); PMID 28713422.

*Supplementary tables containing this information have been added (**Table S2-S4**). We were not able to provide the exact data on censored animals. As is, the amount of pathogenesis and longevity assays often resulted in 6+ hours of scoring daily (and this is focusing on dead worms only). Including scoring living worms would result in doubling this time. However, when we first place worms onto agar plates, we have a rough accounting of the number of worms added. We compared the number of worms scored (those counted as dead during the time course + alive at the last day of scoring). If these two numbers differed by more than 10%, then the experiment is removed from consideration. This is customary in these assays as a measure of ensuring that a large number of worms have not left the agar and escaped analysis, which can introduce artifacts and compromise the interpretation. Typically, though, the number of "lost" / "censored" worms in our experiments is between 5 and 10% (of worms, not replicates).The statistical analysis performed for each of the survival assays is the log-rank test, which is a standard survival distribution comparison for the field (9-13). This test was also used for statistical analysis in the suggested article (14). We performed this analysis by using OASIS, an online application survival analysis tool (10).*

4. The authors should provide more details in the figure legends of the color codes used for their statistical comparisons and what they signify.

The figure legends have been modified to include the meaning of the color codes for statistical comparisons.

5. Figure 2C: the pictures of *tbb-6*::GFP in the absence of stress do not obviously reflect the increase in expression that is reported in the quantification graph for *fib-1*, *nol-56*, and *nol-58* RNAi. Perhaps a higher exposure would allow a better distinction between treatments?

*We understand the reviewer's point and appreciate the benefit of the doubt that he/she has afforded us. However, these conditions were not arbitrarily chosen. Rather, these images were taken with the exposure setting set to ensure that the signal would not be saturated in the spg-7(RNAi) treatment condition. This can be somewhat difficult, as it is ~32-fold higher than the basal Ptbb-6::GFP expression level. Consequently, fib-1(RNAi), nol-56(RNAi), and nol-58(RNAi) (which increased Ptbb-6::GFP expression by a more modest level of ~2-fold) are more difficult to discern. To address this, we used a higher exposure setting for the purpose of ease of visualization and switched to using images only for the basal level (EV(RNAi), **Figure 2C**). We hope that this will make the difference more obvious, but will not misrepresent the difference between these conditions and spg-7(RNAi).*

6. Typo on line 160: "we triggered activation of the of downstream effectors for". Remove "of".

We thank the reviewer for this keen observation. This sentence has been fixed.

7. Typo on line 292-3: “its known regulation by the ATF-7/ATF7” should read “its known regulation by the transcription factor ATF-7/ATF7”.

We again thank the reviewer for their attention to detail. This sentence has been fixed.

Reviewer #2

Mitochondrial surveillance is crucial for maintaining organismal health under various stress conditions, including pathogen infections. In this manuscript entitled “Box C/D Small Nucleolar Ribonucleoproteins Regulate Mitochondrial Surveillance and Innate Immunity”, the authors reported novel functions of the box C/D snoRNA core proteins (snoRNPs) in upregulating mitochondrial surveillance and modulating immune responses. The authors previously reported the roles of Ethanol and Stress Response (ESRE) pathway and ESRE motif in mitochondrial surveillance in response to intracellular stressors. In the current work, the authors found that the box C/D snRNP component proteins, including FIB-1, NOL-56, and NOL-58, upregulated the ESRE pathway and mitochondrial unfolded protein response (UPR^{mt}) under mitochondrial stress conditions. The authors identified the box C/D snRNP component proteins from ESRE motif-binding proteins by using pulldown and mass spec analysis. They showed that knockdown of the box C/D snRNP component proteins upregulated $MAPK^{mt}$ stress pathways. In addition, knocking down the box C/D snoRNPs components upregulated immune effectors and altered the resistance of *C. elegans* against pathogenic bacteria. Overall, the authors suggested that box C/D snoRNPs act as molecular switch between mitochondrial surveillance and innate immunity through ESRE and $MAPK^{mt}$ signaling pathways. This paper starts with the unbiased biochemical identification for ESRE-regulating factors in combination with RNAi screen and ends with molecular genetic analysis for functional importance of the the box C/D snRNP for immunity. This work is thorough and novel and will provide valuable information regarding the research field of mitochondrial biology and immunity. I have basically one major concern in addition to minor ones that will further improve the quality of this excellent paper.

Major comments

1. The genetics in this paper solely depends on RNAi knockdown. RNAi experiments are variable and less reliable than those with mutants, in particular when the data are negative and double RNAi is used. I strongly recommend that the authors strengthen some of the data by performing additional experiments and also by discussing the caveat of using RNAi in the Discussion. Following are my specific suggestions.

1.1. Double RNAi experiments in Figures 2 and 3. The authors performed double RNAi experiments using RNAi-expressing bacteria mixture. They need to show the efficiency of RNAi for the experiments using qRT-PCR (or less preferentially fluorescence reporters) to rule out the possibility that RNAi clones work less efficiently.

We appreciate the reviewer’s concern. Although RNAi is generally very effective in C. elegans, the reviewer is correct that there are occasions when it does not work well. We have ruled out this explanation by quantifying mRNA transcripts of fib-1 and spg-7 in single vs. double knockdown conditions. We observed

indistinguishable levels of knockdown in both single and double knockdowns. This verification has been added as new panels ***Figure S2C*** *and* ***S2D****.*

1.2. Negative data with RNAi in Figures 4, S4 and S7. Here we don't know whether RNAi simply did not work or work less efficiently for these experiments. The authors again need to perform qRT-PCR or reporter assays.

*Again, we appreciate the reviewer's concern. Unfortunately, negative data can sometimes not be unambiguously interpreted. In this case, we have used qRT-PCR (****Figure S5****) to determine that most genes targeted via RNAi have their mRNA levels reduced by at least half compared to EV(RNAi). This is comparable to fib-1 mRNA levels in fib-1(RNAi) condition (both single and double RNAi,* ***Figure S2C****), and is a condition where we have shown that there are clear biological effects.*

1.3. Additional experiments with pmk-1 and atf-7 mutants for Figures 5 and 6. As the mutants are available for pmk-1 and atf-7, and some of the data are the key data, I think they need to test whether the partial and complete requirement of pmk-1 and atf-7 are caused by hypomorphic nature or RNAi.

*We thank the reviewer for the suggestion. We have performed additional qRT-PCR experiments with both pmk-1(km25) and atf-7(gk715) mutants to verify their roles. The loss of atf-7 and pmk-1 significantly reduced the expression of immune genes tested in this paper (****Figure 7E****) in response to fib-1(RNAi), except for irg-1. This is consistent with previous findings (4, 5).*

1.4. Discussion for the limitation of RNAi. Validating RNAi data with the above experiments will dramatically improve the quality of the paper, but practically I also understand that it will require enormous time and efforts. I think the authors may perform some key experiments for validation of RNAi and discuss the limitation of remaining data in the Discussion.

We appreciate the reviewer's concern. Discussion regarding the limitation of RNAi has been added into the Discussion section. Verification of several RNAi knockdowns have also been performed, as noted above.

Minor comments

1. The authors showed the quantification data of GFP fluorescence with representative fluorescent images in Figures 1 and 2. However, they did not display fluorescent images for Figures 3 to 6. It will be better to show fluorescent images of following strains that are not shown in Figures 1 and 2: *Phsp-6*::GFP reporter strains that were crossed with *Patfs-1ΔESRE*::ATFS-1WT in Figure 3A or *Pirg-5*::GFP reporter treated with *fib-1*, *nol-56*, or *nol-58* RNAi in Figure 5.

We thank the reviewer for their suggestion. Representative images for these panels have been added.

2. The central part of the Abstract should be re-written with a little more detail to deliver the main results of this paper better.

We have attempted to revise the abstract to more effectively state our main results. This feedback was very useful.

3. The authors explained three mitochondrial surveillance pathways based on previous studies with various organisms. Those organisms need to be specified in the Introduction.

We appreciate the suggestion, and we have made this change to the Introduction.

4. On page 6, the authors identified regulatory components of the ESRE pathway by using pulldown assays. It will be better for the authors to add a Supplemental table for listing up those 75 candidates that were identified.

*A supplementary table that lists all identified proteins from the pulldown assays has been added (**Table S1**).*

5. In Figure legends, please explain specific information regarding the data with more details. For example, the authors did not explain the meaning of the colors of in the bar graphs for statistical significance at the beginning, although they explained this on page 9.

We have modified all figure legends to incorporate more detailed information.

6. On page 9, in figure 3, they may want to move Figure S1B to Figure 3B for better comparison between the effects of RNAi starting at L3 and starting at L1.

*We understand and appreciate the suggestion. We have moved this panel to **Figure 3A**.*

7. Bar graphs in the Figures will be better to be changed with dot plots. That is a standard these days.

All bar graphs have been switched into box plots with points.

8. On page 12, figure 5, the authors showed the expression of *Pirg-1*::GFP and *Pirg-5*::GFP reporters to show the effects of Box C/D snoRNPs on innate immune responses. It will be more convincing if the authors show more targets for further verification, using additional reporters or by performing quantitative RT-PCR.

As discussed in our response to Reviewer #1 (point 4.a), we have performed qRT-PCR experiments to verify that the knockdown of Box C/D snoRNPs increases host's immune response. Furthermore, we also assayed

the expression of an immune reporter strain carrying $P_{T24B8.5}$::GFP (new panel as ***Figure 6D****), and observed similar results to Pirg-5::GFP expression.*

9. On page 14, the authors mentioned that RNAi targeting box C/D snoRNPs components reduced the survival of worms in liquid-based pathogen killing assay, whereas the knockdown increased survival of worms in slow killing assay on agar plates. The authors need to elaborate the conclusion and add discussion for these seemingly contradicting data. The authors also need to change the subheading of this paragraph because it is confusing with respect to the main point of this paper mentioned in the Abstract.

We understand the reviewer's point. Generally speaking, the differences between these assays can be relatively nuanced and are sometimes of less interest to a wider audience (we believe that they are a consequence of different physiological consequences of infection, which takes place in different ways for solid and liquid conditions). However, we do appreciate that to many reviewers, this difference will seem confusing. We have attempted to provide a brief but meaningful explanation in the Discussion section. We have also modified the subheading of this paragraph, which now reads: Box C/D snoRNPs provide protection against mitochondrial damage from pathogens

10. In Discussion section, the authors suggested a hypothesis that modification on rRNA by box C/D snoRNP may facilitate translation of specific sub-population of transcripts. A previous paper (Liberman et al., 2020, Science advances; DOI: 10.1126/sciadv.aaz4370) reported that ribosome methylation can facilitate selective translation. I suggest the authors cite this paper in the Discussion section to support the hypothesis.

This paper has been added to the Discussion section as suggested.

11. As they mentioned in the Introduction, many papers including Pellegrino et al. 2014, Nature; Kirienko et al. 2015, PNAS; Jeong et al. 2017, EMBO J; Deng et al. 2019, PNAS; Campos et al., 2021 EMBO Rep. have shown that mitochondrial surveillance pathways such as UPRMT activates innate immune responses in *C. elegans*. They need to cite missing literature and discuss the effects of snoRNPs on protective roles of mitochondrial surveillance systems upon pathogen exposure.

Citations for these articles along with discussion on the roles of mitochondrial surveillance systems have been added into the Discussion section.

12. For describing Figure 1E, Tiku et al. 2017 needs to be cited and mentioned, because they reported that *fib-1* RNAi extends lifespan.

The citation and mention for Tiku et al. 2017 has been added for this panel.

13. Please unify the units (e.g. use 'hours' instead of 'h' in the method section).

All units have been unified. Thank you for the suggestion.

14. Please explain each abbreviation in Figure legends and Methods (on page 22, e.g., SK).

Explanations on each abbreviation have been added into Figure legends and Methods.

Reviewer #3

This works examines the very interesting but complex interactions among various mitochondrial surveillance pathways including mtUPR, MAPK, and ethanol and stress response network (ESRE) in *C. elegans*. Here, the authors seek to unravel the transcriptional regulation of the ESRE network and understand the various interacting pathways. Through biochemical mass spec screens, the authors find surprisingly that components of Box C/D SnoRNA methylation complex physically associate with an 11 bp DNA motif comprising the ESRE element. This is unexpected because Box C/D SnoRNA complex is mainly involved in the processing rRNA. Consistent with a regulatory role, knockdown of these components dampens the induction of an ESRE element reporter, and correspondingly the mtUPR reporter *hsp-6*, but not the mito mapk reporter *tbb-6*, suggesting specific interaction. In line with specificity, knockdown of the HACA SnoRNA pseudouridylation complex has no such effects. Nor is it simply due to reduced translation, since kd of translational regulators does not inhibit ESRE induction. Further the authors find that knockdown of C/D SNO complex triggers expression of innate immune signaling genes in a manner dependent on the atf-7 transcription factor. Corresponding, worms are protected in slow killing, but not fast killing assays of *P. aeruginosa*.

This paper represents a conceptual advance as it suggests potential tradeoffs between mitochondrial surveillance and innate immune response, but there are several technical deficiencies that need to be addressed.

1. There is little mechanistic insight into how fibrillarin and nol-56 regulates the ESRE site either directly or indirectly, though the authors remark extensively on this in the discussion. Are the authors suggesting that fib-1 or nol-56 directly bind directly to the ESRE element and act in transcriptional regulation? Or is this a non-specific effect of an abundant protein binding to nucleic acid? To address this, they should also compare wt and mutated ESRE site with worm extracts. Notably, the gel shift in Figure 1 is not publication quality.
*We appreciate the reviewer's concerns regarding the mechanistic insight of ESRE site regulation. We favor the possibility that the Box C/D snoRNPs pair with one or more unidentified C/D snoRNAs that match the ESRE consensus site. However, we obviously have not identified the potential snoRNA yet. Additionally, we have performed an EMSA experiment with mutated ESRE sites (three mutations per element), in which we observed the reduction of 'Shift', as compared to the wild type ESRE baits. We understand the reviewer's critique of the gel shift figure. However, despite extensive efforts, we have been unable to obtain better figure data in the past. As we had a number of new experiments that needed to be done for these revisions, we have focused on those rather than repeating EMSA. We have added data on the mutant ESRE sequence as a supplemental figure **Figure S1**.*

2. Reporter constructs can be misleading, particularly if they are multicopy arrays. Can the authors confirm that kd of Box C/D components regulates expression at the level of mRNA of a number of ESRE element containing inducible genes?

*We appreciate the reviewer's concern. To confirm that the knockdown of Box C/D components indeed decreases the expressions of ESRE-containing genes, we performed additional qRT-PCR experiments. N2 worms were reared on EV(RNAi) or fib-1(RNAi) and then treated for 8 hours with rotenone (to induce ESRE expression) or DMSO control. Worms that were reared on fib-1(RNAi) had significantly lower ESRE genes expression. This panel has been added as **Figure 1G**.*

3. It has been previously shown that knockdown of nucleolar proteins (*fib-1*, and *nol-6*) result in enhanced pathogen resistance, so the connection innate immunity to nucleolar function aspect is not entirely novel. However, extending these observations to the whole C/D SnoRNA complex is interesting, and appears specific since similar physiology is not seen with HACA SnoRNA and pseudouridylation. The question is whether this contrast reflects a difference in the degree of RNAi knockdown, however, and thus levels of transcripts should be quantitated by RT-PCR.

*We appreciate the reviewer's concern and has correspondingly performed transcript quantification via qRT-PCR (**Figure S5**). We observed reduction of mRNA transcripts to a comparable level to fib-1 mRNA levels in fib-1(RNAi) condition.*

4. Opposite to Box C/D knockdown, the authors nicely show that kd of a number of translational regulators still support activation of the ESRE reporter. Is this also true of small and large subunits of the cytosolic ribosome?

The reviewer pointed out an intriguing point. To address this point, we selected seven random rpl and rps genes and knocked them down via RNAi. Unfortunately, the knockdown of all selected small and large subunits of the cytosolic ribosome greatly impacted on worm physiology, even when RNAi was started at L3 (as compared to Box C/D snoRNPs knockdown that does not significantly alter worms' size when RNAi was performed at L3). This makes it difficult or impossible to meaningfully compare the conditions (i.e., it is not clear that doing the RNAi later would achieve sufficient knockdown to meaningfully compare to empty vectors, and an absence of outcome could therefore not be meaningfully interpreted.) This panel will not be included in the article, but is attached below.

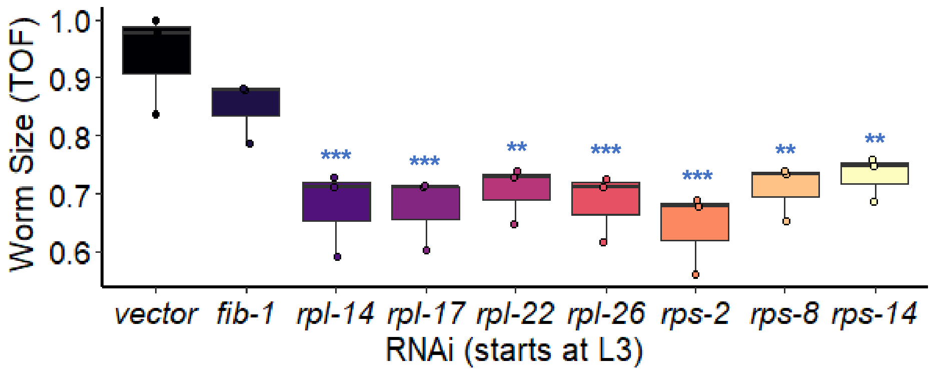

5. A major technical problem could arise from the use of double RNAi. Double RNAi often causes mutual hindrance of the knockdown, and could thus appear as suppression. Empty vector control is not the best one. Instead, the authors should use luciferase RNAi, in which the RNAi machinery is induced, but is absent a target, in order to validate their findings in at least a few critical experiments.

We thank the reviewer for the suggestion, and are familiar with this outcome. In full disclosure, this appears to be a more complicated situation, as double RNAi sometimes appears to work just fine and other times does not (speaking across labs and assays, not specifically in our lab).

However, we obtained a luciferase(RNAi)-expressing E. coli from the laboratory of Dr. Antebi. We tested the effect of luciferase(RNAi) as compared to vector(RNAi) on 3XESRE::GFP-expressing worms, and found that there was no significant difference between empty vector and the luciferase control. We also tested the effect of luciferase(RNAi) vs. vector(RNAi) in several double RNAi settings and found no significant difference. These panels have been added as ***Figures S2A-S2B****.*

6. Figure 3 is confusing and not adequately explained. The authors delete the ESRE element from the ATFS-1 promoter and claim that it has lower levels of *hsp-6* expression upon rotenone exposure. Is this a crispr deletion in the chromosome? Judging from the methods it looks like a transgenic insertion. What is it compared to? A wt multicopy array? How can the authors really compare a wt multi copy array to a mutant one without knowing copy number?

As the reviewer suspects, the strain carrying the atfs-1 mutant missing the ESRE site in its promoter was generated via CRISPR. This strain was then crossed to the worm strain carrying the Phsp-6::GFP reporter. Worms were homozygosed at the atfs-1 locus to ensure the absence of the ESRE site in the promoter (as confirmed by PCR). This led to the generation of a strain that is Patfs-1$^{\Delta ESRE}$::ATFS-1^{WT}; Phsp-6::GFP. Expression of GFP in this strain is then compared to a strain where the atfs-1 locus includes the ESRE site. This allows the effect of the ESRE site in regulation of hsp-6 to be evaluated. We hope that this has been made more clear in the revised version of the paper.

7. Overall the paper suggests that snoRNPs are required for activation of mitochondrial stress response ESRE as well as UPR^{mt}, but snoRNP complex was not required for activation of MAPK^{mt} response. Therefore, I don't understand the model, as it shows UPR^{mt} suppresses MAPK^{mt} (after stress), yet *spg-7* RNAi activates both UPR^{mt} as well as MAPK^{mt}, so there should be an activation arrow rather than suppression. Further, there is a clear requirement of snoRNP for UPR^{mt} activation, but it has no effect at all on MAPK^{mt} activation, again not justified by their model.

We thank the reviewer for their keen insight here. During the revision process, we realized that this model could lead to confusion, and we have thus modified it. To be more clear, we previously found that the UPR^{mt} ***suppressed*** *the* ***basal*** *expression of MAPK^{mt}. Consistent with this, disruption of atfs-1 by RNAi* ***increased basal*** *Ptbb-6::GFP expression (7). However, atfs-1(RNAi) also* ***increased*** *Ptbb-6::GFP expression*

*under **induced** conditions (e.g., spg-7(RNAi) treatment) (15). These observations led us to hypothesize that ATFS-1 may act to limit unnecessary MAPK^{mt} expression under normal conditions to prevent inappropriate stress responses. During stress, however, this repression may be lifted to allow MAPK^{mt} to help restore cellular health.*

*Since box C/D snoRNPs are necessary for normal function of ATFS-1, their disruption would lead to **increased basal** Ptbb-6::GFP expression, much as would be seen by atfs-1(RNAi). Under stress conditions, however, the MAPK^{mt} response appears much less dependent upon ATFS-1 level, so it would be similarly independent of ESRE and the box C/D snoRNPs, since our only observation of these factors regulating the MAPK^{mt} pathway is via ATFS-1 function.*

1. Van Raamsdonk J, Hekimi S. FUdR causes a twofold increase in the lifespan of the mitochondrial mutant gas-1. Mechanisms of ageing and development. 2011;132(10):519-21.
2. Aitlhadj L, Stürzenbaum S. The use of FUdR can cause prolonged longevity in mutant nematodes. Mechanisms of ageing and development. 2010;131(5):364-5.
3. Kato Y, Miyaji M, Zhang-Akiyama Q. FUdR extends the lifespan of the short-lived AP endonuclease mutant in Caenorhabditis elegans in a fertility-dependent manner. Genes & genetic systems. 2017;91(4):201-7.
4. Campos J, Wu Z, Rudich P, Soo S, Mistry M, Ferreira J, et al. Mild mitochondrial impairment enhances innate immunity and longevity through ATFS-1 and p38 signaling. EMBO reports. 2021;22(12):e52964.
5. Estes KA, Dunbar TL, Powell JR, Ausubel FM, Troemel ER. bZIP transcription factor zip-2 mediates an early response to Pseudomonas aeruginosa infection in Caenorhabditis elegans. Proceedings of the National Academy of Sciences. 2010;107(5):2153-8.
6. Holley CL, Li MW, Scruggs BS, Matkovich SJ, Ory DS, Schaffer JE. Cytosolic Accumulation of Small Nucleolar RNAs (snoRNAs) Is Dynamically Regulated by NADPH Oxidase. Journal of Biological Chemistry. 2015;290(18):11741-8.
7. Tjahjono E, McAnena AP, Kirienko NV. The evolutionarily conserved ESRE stress response network is activated by ROS and mitochondrial damage. BMC Biology. 2020;18(1):74.
8. Tiku V, Kew C, Mehrotra P, Ganesan R, Robinson N, Antebi A. Nucleolar fibrillarin is an evolutionarily conserved regulator of bacterial pathogen resistance. Nature Communications. 2018;9(1):3607.
9. Park H, Jung Y, Lee S. Survival assays using Caenorhabditis elegans. Molecules and cells. 2017;40(2):90-9.
10. Yang J, Nam H, Seo M, Han S, Choi Y, Nam H, et al. OASIS: online application for the survival analysis of lifespan assays performed in aging research. PloS one. 2011;6(8):e23525.
11. Stroustrup N, Ulmschneider B, Nash Z, López-Moyado I, Apfeld J, Fontana W. The Caenorhabditis elegans Lifespan Machine. Nature methods. 2013;10(7):665-70.
12. Win M, Yamamoto Y, Munesue S, Han D, Harada S, Yamamoto H. Validated Liquid Culture Monitoring System for Lifespan Extension of Caenorhabditis elegans through Genetic and Dietary Manipulations. Aging and disease. 2013;4(4):178-85.
13. Zhao Y, Gilliat A, Ziehm M, Turmaine M, Wang H, Ezcurra M, et al. Two forms of death in ageing Caenorhabditis elegans. Nature communications. 2017;8:15458.
14. Petrascheck M, Miller D. Computational Analysis of Lifespan Experiment Reproducibility. Frontiers in genetics. 2017;8:92.

15. Munkácsy E, Khan MH, Lane RK, Borror MB, Park JH, Bokov AF, et al. DLK-1, SEK-3 and PMK-3 Are Required for the Life Extension Induced by Mitochondrial Bioenergetic Disruption in C. elegans. PLOS Genetics. 2016;12(7):e1006133.
